# Synthesis and Biological Activity of 6-Selenocaffeine: Potential Modulator of Chemotherapeutic Drugs in Breast Cancer Cells

**DOI:** 10.3390/molecules18055251

**Published:** 2013-05-08

**Authors:** Inês L. Martins, Joana P. Miranda, Nuno G. Oliveira, Ana S. Fernandes, Sandrina Gonçalves, Alexandra M. M. Antunes

**Affiliations:** 1 Centro de Química Estrutural, Instituto Superior Técnico, Universidade Técnica de Lisboa, 1049-001 Lisboa, Portugal; E-Mail: ines.l.martins@ist.utl.pt; 2 Research Institute for Medicines and Pharmaceutical Sciences (iMed.UL), Faculty of Pharmacy, University of Lisbon, Av. Prof. Gama Pinto, 1649-003 Lisbon, Portugal; E-Mails: jmiranda@ff.ul.pt (J.P.M.); ngoliveira@ff.ul.pt (N.G.O.); sandrinagoncalves@campus.ul.pt (S.G.); 3 CBIOS, Universidade Lusófona de Humanidades e Tecnologias, 1749-024 Lisboa, Portugal; E-Mail: ana.fernandes@ulusofona.pt

**Keywords:** organoselenium compounds, caffeine, modulator of the cytoxicity, oxaliplatin, doxorubicin, cancer therapy

## Abstract

We report the development of a new microwave-based synthetic methodology mediated by Woollins’ reagent that allowed an efficient conversion of caffeine into 6-selenocaffeine. A preliminary evaluation on the modulation of antioxidant activity upon selenation of caffeine, using the DPPH assay, indicated a mild antioxidant activity for 6-selenocaffeine, contrasting with caffeine, that exhibited no antioxidant activity under the same experimental conditions. Interestingly, whereas 6-selenocaffeine has revealed to have a low cytotoxic potential in both MCF10A and MCF-7 breast cells (24 h, up to 100 µM, MTT assay), a differential effect was observed when used in combination with the anticancer agents doxorubicin and oxaliplatin in MCF-7 breast cancer cells. The co-treatment of doxorubicin (1 µM) and 6-selenocaffeine (100 µM) resulted in a slight decrease in cellular viability when compared to doxorubicin (1 µM) alone. Conversely, the seleno-caffeine derivative at the same concentration markedly increased the viability of oxaliplatin (100 µM)-treated cells (*p* < 0.01). Overall, this work highlights an emerging methodology to synthesize organoselenium compounds and points out the differential roles of 6-selenocaffeine in the modulation of the cytotoxicity of anticancer agents.

## 1. Introduction

The considerable number of reports of organoselenium compounds presenting antineoplastic effects in recent years has markedly increased the interest in this class of compounds [1,2,3,4,5,6,7,8,9,10]. Organoselenium compounds are thus emerging as promising downstream candidates for cancer therapy due to their ability to modulate multiple physiological functions implicated in cancer development, presenting either antioxidant [11,12] anticancer / chemopreventive [13,14] or apoptotic activities [15]. 

**Figure 1:**
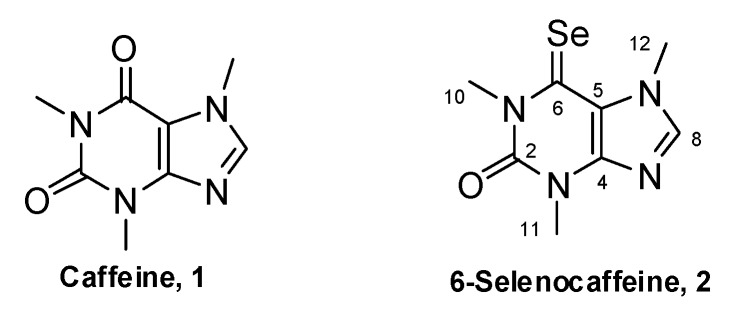
Structures of caffeine (**1**) and its selenium analogue, 6-selenocaffeine (**2**).

Caffeine [1,3,7-trimethyl-1*H*-purine-2,6-(3*H*,7*H*)-dione, 1, Figure 1] is a natural occurring methylxanthine. Its frequent and common usage as component of tea, coffee and soft drinks, has lead researchers to study intensively its biological properties. In particular, the antioxidant activity [16] and the effects in cell cycle and cancer have been highly investigated [17]. Despite the interest of some of the reported properties a common feature is the usually high concentrations of caffeine required. Indeed, some of these properties are not achievable without serious adverse effects. Nonetheless, the basic caffeine scaffold is of unquestionable interest as lead compound for the development of new derivatives with enhanced activities and/or lower toxicities. Hence, considering both the beneficial effects of the selenium-containing compounds and caffeine, the synthesis of a selenium derivative was carried out by replacing the oxygen of a carbonyl group by a selenium atom. Towards this goal, a new synthetic microwave-based methodology to prepare the 6-selenocaffeine derivative **2** (Figure 1) from caffeine was developed and the synthesized compound was structurally characterized. Additionally, this report also focuses on the evaluation of the antioxidant potential of the novel 6-selenocaffeine derivative, as well as on the assessment of its cytotoxicity profile in human mammary cells of non-tumor origin, using MCF10A cells. The potential of 6-selenocaffeine to modulate the cytotoxicity of standard anticancer drugs was also addressed using MCF7 human breast cancer cells. In this context, 6-selenocaffeine was evaluated in combination with doxorubicin and oxaliplatin, two drugs belonging to distinct chemotherapeutic groups.

## 2. Results and Discussion

### 2.1. Synthesis and Structural Characterization of 6-Selenocaffeine

Examples of interconversion of carbonyl into selenocarbonyl moieties are relative scarce in the literature, mostly due to the lack of effective synthetic procedures for this transformation. Nonetheless, among the selenium reagents available for this group interconversion [18], the Woollins’ reagent (2,4-diphenyl-1,3-diselenadiphosphetane-2,4-diselenide, WR), a selenium analogue of Lawesson’s reagent [19], presents important advantages, namely its higher stability in air and the availability of an easy method for its preparation [20]. Thus, the use of this reagent for the conversion of carbonyl groups to selenocarbonyl groups in the synthesis of selenoamides [21] combined with the success of the microwave-accelerated Lawesson’s reagent-mediated thionation of flavonoids [22,23] motivated us to test caffeine selenation using WR under microwave irradiation. Screening reactions were conducted with different solvents and quantities of WR. Different conditions of temperature, microwave potency and time of microwave irradiation were also tested (Table 1).

**Table 1:** Experimental conditions used for the optimization of caffeine selenation with WR under microwave irradiation.

WR (µmol)	Solvent	Irradiation Time (min)	Max. Temp. (°C)	Irradiation potency (W)	2 ɳ (%)
103	acetonitrile	5	150	175	4
206		5	150	175	n.r. ^a^
103		5	130	200	n.r. ^a^
206		10	130	200	n.r. ^a^
103		5	130	175	2
103		5	150	250	3
103		10	150	250	5
103		5	170	300	2
103	toluene	50	170	300	7
103		90	170	300	17
206		180	170	300	23
206		180	170	300	30
156	*p*-xylene	180	170	300	42
206	1,4-dioxane	180	170	300	19
206	propionitrile	180	170	300	21

^a^ n.r. no reaction.

The optimized conditions (Table 1, highlighted in grey) consisted of 180 min. of microwave irradiation at 300 W (170 °C) of a *p*-xylene solution of WR (156 µmol) and caffeine (257 µmol), in a sealed Pyrex microwave vial. Under these conditions the 6-selenocaffeine derivative was selectively obtained in 42% yield. These experimental conditions were also successfully applied for the selenation of the pyrimidine derivative uracil (see Experimental section), which anticipates the general character of the developed methodology. Of note is the fact that when conventional heating was used, under similar experimental conditions, selenocaffeine derivative **2** was obtained in only 2% yield; as a clear indication that microwave irradiation improves the yield of caffeine selenation mediated by WR. 

Evidence for the formation of selenated derivative was first obtained by mass spectrometry. In fact, the low and high resolution mass spectra, obtained either by electrospray ionization or electronic impact, showed indistinctly five signals corresponding to selenium isotopes with the expected *m/z* values for the protonated molecules and molecular ions of **2**, respectively. Further crucial evidence for the existence of a selenocarbonyl group (C=Se) was provided by ^77^Se-NMR, where a signal at 600.5 ppm was obtained (*cf.* Supplementary Information), which is in agreement with the ^77^Se-NMR chemical shifts of C=Se groups reported in the literature [24]. The assignment of all ^1^H-NMR and ^13^C-NMR resonances was based on the correlations observed in both HSQC and HMBC spectra. The most noticeable differences in the NMR spectra were the downfield shifts of signals corresponding to positions C5, C6 and C10 (Table 2) in 6-selenocaffeine, as compared with caffeine. Indeed the low field chemical shift of the selenocarbonyl carbon atom was already expected [25].

**Table 2:** Comparison of ^1^H and ^13^C-NMR resonances in caffeine (**1**) with 6-selenocaffeine (**2**).

Carbon	Caffeine (1)		6-Selenocaffeine (2)	
	^1^H-NMR ^a^	^13^C-NMR ^a^	^1^H-NMR ^a^	^13^C-NMR ^a^
	δ (ppm)	δ (ppm)	δ (ppm)	δ (ppm)
2	---	150.9	---	149.1
4	---	148.0	---	143.8
5	---	106.5	---	121.2
6	---	154.4	---	175.8
8	7.97	142.7	8.35	147.4
10	3.12	27.4	3.75	37.4
11	3.36	29.3	*c.a.* 3.38	30.3
12	3.84	33.1	4.10	35.8

^a^ The spectra were recorded in DMSO-d_6_ at room temperature.

**Figure 2:**
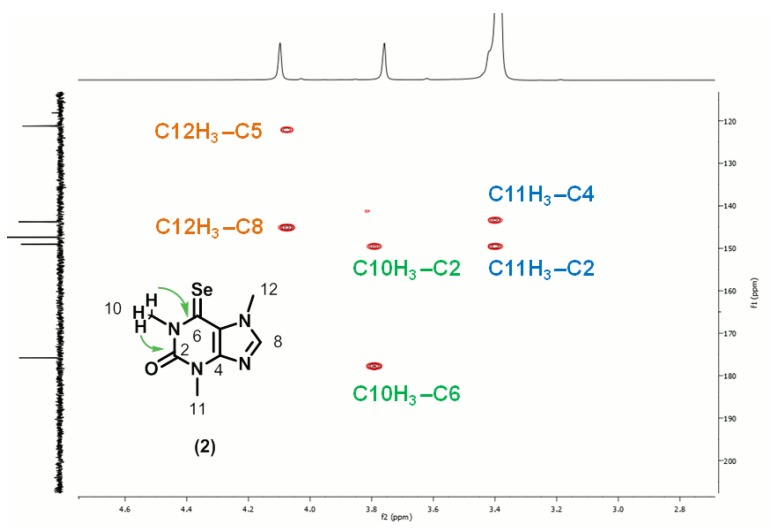
Expanded region of the ^1^H- ^13^C-HMBC spectrum of 6-selenocaffeine (**2**), displaying the 3-bond connectivities between the methyl protons at position C10 with quaternary carbons C2 and C6.

The assignment of C6 as the position of selenation was based on the ^1^H-^13^C three bond correlations observed in the HMBC spectra (Figure 2) between the methyl protons at position C10 and C11 and quaternary carbons: protons of both methyl groups presented correlations with the carbonyl carbon C2 (149.1 ppm); but whereas the C11-H_3_ (*ca.* 3.38 ppm) protons presented a correlation with C4 (143.8), the C10-H_3_ (3.75 ppm) protons presented correlations with the low field selenocarbonyl carbon C6 (175.8 ppm). 

### 2.2. Antioxidant Activity of 6-Selenocaffeine (**2**)

**Figure 3:**
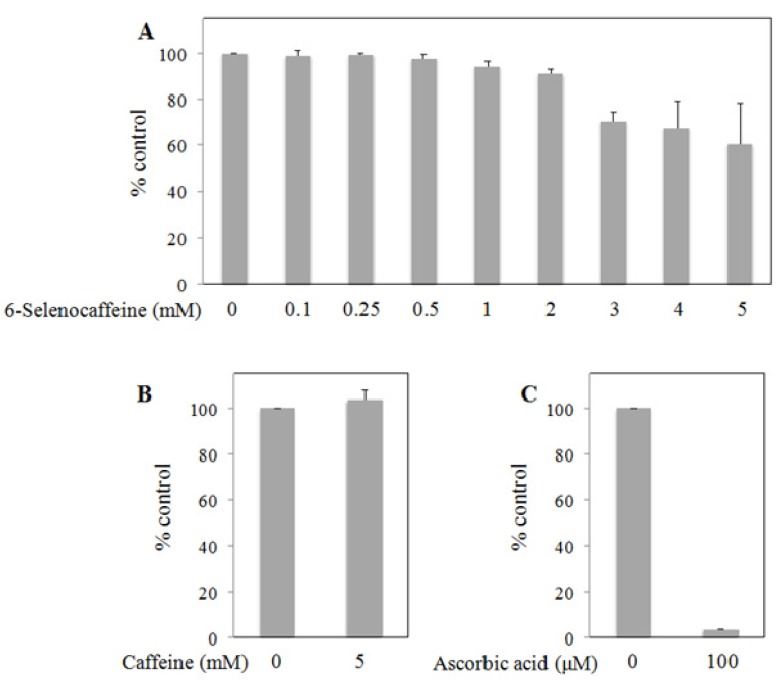
Scavenging effect of DPPH radical by 6-selenocaffeine (**A**), caffeine (**B**), and ascorbic acid (**C**) Results are expressed as mean ± SD of two independent experiments and were calculated considering the absorbance of the negative control (DPPH 75 μM in ethanol) as 100%.

Whereas caffeine has been suggested as an antioxidant [26], some studies demonstrate an absence of antioxidant properties [27,28] or even prooxidant effects [26,29]. In addition, using DPPH-based assays, different authors have also reported absence of antioxidant activity for caffeine [27,28,29]. However, the antioxidant properties of organoselenium compounds are often argued as support of the potential pharmacological applications of this class of compounds. Therefore, a preliminary study on the modulation of the antioxidant activity upon replacement of the carbonylic O atom of caffeine by its chalcolgen analogue Se was carried out using the DPPH assay (Figure 3). Our data evidenced an improvement of the antioxidant effect upon selenation of caffeine, albeit the scavenging capacity of 6-selenocaffeine is only moderate. Specifically, we have observed for 6-selenocaffeine that: (1) up to 2.0 mM the antioxidant activity of this seleno-derivative was not evident; (2) this compound at 3 mM decreased the amount of DPPH to approximately 70% (*p* < 0.05) of the negative control (Figure 3A); (3) for the higher concentrations tested (4 and 5 mM) the decrease in the amount of DPPH was in the same range as with 3.0 mM 6-selenocaffeine. Conversely, when caffeine was tested under the same experimental conditions, no antioxidant effects were observed at 5 mM concentration (Figure 3B). Very high caffeine concentrations (10 and 15 mM) demonstrated no antioxidant effect as well (data not shown). Ascorbic acid, a well-recognized antioxidant, was used as positive control. As expected, this compound showed a marked scavenging activity decreasing the amount of DPPH to 3.6% of the negative control (Figure 3C; *p* < 0.001). 

Taking into consideration that protection against peroxides/peroxinitrite, glutathione peroxidase-like activity and metal-binding capacity are also frequent antioxidant mechanisms among organoselenium compounds [12,30,31,32], other antioxidant modes of action of 6-selenocaffeine should be further exploited in future work. 

### 2.3. Assessment of the Cytotoxicity Profile of 6-Selenocaffeine (**2**) in Breast Cells

**Figure 4:**
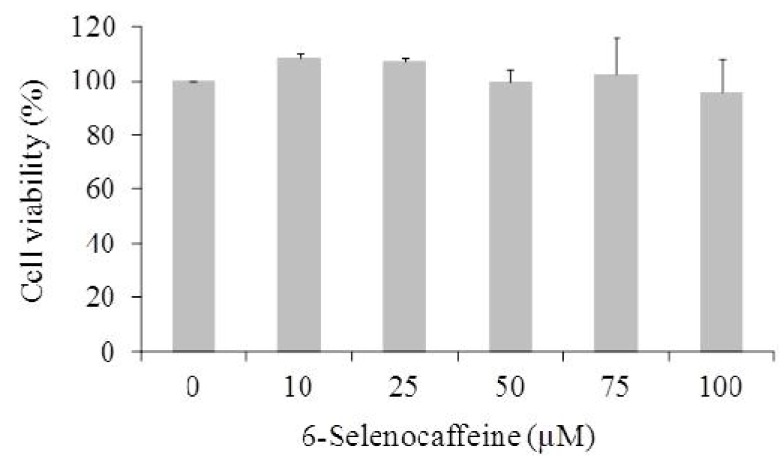
Cell viability of MCF10A cells treated with different concentrations of 6-selenocaffeine as evaluated by the MTT assay (24 h). Values represent mean ± SD of at least two independent experiments, and are expressed as percentages relative to non-treated control cells.

The next step of this work was to assess the cytotoxicity profile of 6-selenocaffeine in mammalian cells (Figure 4). For this purpose the seleno compound was tested in the MCF10A cell line using the MTT assay. This non-tumor human breast epithelial cell line is widely used and it may be considered representative of “normal-type” mammary cells. In addition, the MTT assay has also been thoroughly used by several authors for the assessment of the cytotoxicity of chemicals. This assay is a tetrazolium reduction-based methodology, providing therefore a measure of cell viability in terms of mitochondrial function [33]. In this work, different concentrations of 6-selenocaffeine up to 100 µM were evaluated in a standard 24 hour incubation protocol in MCF10A cells (Figure 4). The results showed that this compound displayed cell viability values similar to untreated control cells revealing no clear cytotoxicity potential to the MCF10A cells under the experimental conditions tested.

### 2.4. 6-Selenocaffeine (**2**) as a Possible Modulator of the Cytotoxicity Induced by Chemotherapeutic Drugs

Selenium has been suggested as a potential agent to be used not only in cancer prevention, but also in cancer treatment, where in combination with anticancer drugs or ionizing radiation, it can improve the efficacy of anticancer therapy [34]. In view of this we aimed to evaluate whether 6-selenocaffeine could indeed be effective on the modulation of the cytotoxic potential of doxorubicin and oxaliplatin in MCF-7 human breast cancer cells. Both drugs are used in many protocols for cancer treatment. Doxorubicin is widely used for a plethora of human malignancies, being very important in breast cancer therapy [35,36]. This anthracyclin promotes DNA intercalation, binding and alkylation, interferes with helicase and topoisomerase II activity, induces apoptosis, and generates reactive oxygen species (ROS) [37]. Oxaliplatin is a platinum-based drug that mainly forms intra-strand cross-links, disrupting DNA replication and transcription. ROS generation has also been described for oxaliplatin [38]. Although oxaliplatin is commonly used in combination with 5-fluorouracil/leucovorin to treat advanced colorectal cancer, recent reports have also suggested its use in metastatic breast cancer [38].

**Figure 5:**
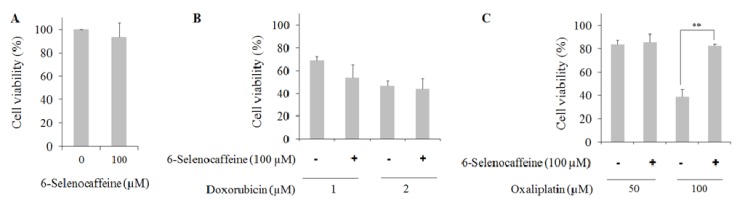
Cell viability of MCF7 cells exposed to doxorubicin and oxaliplatin (24 h incubation) in the presence of 6-selenocaffeine as evaluated by the MTT assay. Values represent mean ± SD and are expressed as percentages relative to non-treated control cells. (**A**) Effect of 6-selenocaffeine on the viability of cells treated for 24 h (n = 6); (**B**) Effect of 6-selenocaffeine on the viability of cells treated with doxorubicin for 24 h (n = 3); (**C**) Effect of 6-selenocaffeine on the viability of cells treated with oxaliplatin for 24 h (n = 3) (** *p* < 0.01 when compared with MCF7 cells treated only with oxaliplatin).

In the present study the effect of 6-selenocaffeine (100 µM) was evaluated using the MTT assay towards two concentrations of doxorubicin (1 and 2 µM) or oxaliplatin (50 and 100 µM), representative of two distinct cytotoxicity levels. The results from these experiments are depicted in Figure 5. The effect of 6-selenocaffeine alone is first presented in Figure 5A. This selenium derivative at 100 µM concentration did not show a relevant cytotoxic effect in MCF7 cells (N.S.). The viability results are also similar to those presented by 6-selenocaffeine in non-tumor MCF10A cells at the same concentration level (100 µM, Figure 4). Regarding the combined effect of 6-selenocaffeine with the chemotherapeutic drugs, two distinct patterns were observed. For doxorubicin, at 1 µM a slight, non- significant, decrease in cell viability was observed upon 6-selenocaffeine treatment. This effect was not observed with doxorubicin 2 µM (Figure 5B). In contrast, for oxaliplatin (100 µM) a significant increase in cell viability from 37% to more than 80% (*p* < 0.01) was observed in cells co-treated with 6-selenocaffeine when compared with oxaliplatin alone (Figure 5C). This marked protection afforded by 6-selenocaffeine was however not observed for the lower oxaliplatin concentration tested (50 µM), showing that this response may be concentration-related, being notorious only for a major toxic insult.

The reason for the distinct responses observed for doxorubicin and oxaliplatin is not known, although this may be due to the intrinsic mechanisms of action of the abovementioned drugs or, alternatively, it may be a consequence of the possible interactions of 6-selenocaffeine with DNA, rendering differential outcomes for both drugs. 

Some authors have hypothesized that caffeine could interfere with intercalating anticancer drugs like doxorubicin by forming π-π molecular complexes with the drug, thereby blocking the planar aromatic drugs from intercalating into the DNA and ultimately lowering the toxicity of the drug to the cancer cells [39]. However, our results suggest a differential effect upon caffeine selenation. Hence, if this type of interaction was present also with 6-selenocaffeine, the outcome would likely be a decrease in doxorubicin cytotoxicity, rather than the slight cytotoxic effect observed in our work (Figure 5B). 

Regardless of the possible underlying mechanisms triggered by 6-selenocaffeine, the notable reduction of the cytotoxicity of oxaliplatin (100 µM) is an interesting finding of this report. While ensuring the oxaliplatin therapeutic efficacy, the protective effect afforded by 6-selenocaffeine could be further exploited in a different approach for the mitigation of oxaliplatin side effects, for instance in terms of the dose-limiting neurotoxicity. 

## 3. Experimental

### 3.1. Chemicals

Caffeine was purchased from BDH Chemicals Ltd. (Kingston upon Hull, UK) and used without further purification. The Woollins’ reagent (WR) was prepared as described in Wood *et al. *[40] involving initial formation of the pentamer (PhP)_5_. All other reagents and solvents used in 6-selenocaffeine synthesis were obtained from Sigma-Aldrich Química, S.A (Madrid, Spain) and used as received. DPPH (1,1-diphenyl-2-picrylhydrazyl radical), ascorbic acid, phosphate buffered saline (PBS) 0.01 M (pH 7.4), trypsin, DMEM, DMEM/Nutrient Mixture F-12 Ham (DMEM/F12), penicillin-streptomycin solution, horse serum, foetal bovine serum (FBS), insulin from bovine pancreas, hydrocortisone, cholera toxin, human epidermal growth factor (EGF), thiazolyl blue tetrazolium bromide (MTT) were purchased from Sigma-Aldrich. Dimethylsulfoxide (DMSO) and absolute ethanol were obtained from Merck (Darmstadt, Germany).

### 3.2. Instrumentation

Microwave syntheses were performed on a CEM Discover^®^ BenchMate microwave reactor (CEM Microwave Technology Ltd., Buckingham, UK). Experiments were performed in sealed Pyrex microwave vials (300 W maximum power) using temperature control mode. Melting temperatures were measured in a Leica Galen III hot stage apparatus and are uncorrected. The UV measurements were recorded on a Perkin Elmer Lambda 35 UV/VIS spectrophotometer. Infrared (IR) spectra were recorded on a Perkin-Elmer 683 IR spectrometer (Waltham, MA, USA); group frequencies are reported in cm^−1^. The sample purity was assessed by HPLC-DAD analysis conducted on an Ultimate 3000 Dionex system (Dionex Co., Sunnyvale, CA, USA) with a Luna C18 (2) column (250 mm × 4.6 mm; 5 mm; Phenomenex, Torrance, CA, USA), at a flow rate of 1 mL min^−1^. The UV absorbance was monitored at 254 nm. A 30-min linear gradient from 5 to 70% acetonitrile in 0.1% aqueous formic acid, followed by a 2-min linear gradient to 100% acetonitrile and an 5-min isocratic elution with acetonitrile, was used. Low resolution mass spectra were performed with a Varian system consisting of a 500-MS ion trap mass spectrometer, with an ESI ion source (Varian, Inc., Palo Alto, CA, USA). High resolution mass spectra were obtained on a Finnigan FT/MS 2001-DT spectrometer (ThermoScientific, Madrid, Spain) operated in the electronic impact ionization mode. ^1^H-NMR spectra were recorded on Bruker Avance III 500 spectrometers (Bruker BioSpin GmbH, Rheinstetten, Germany) operating at 500 MHz. ^13^C-NMR spectra were recorded on the same instrument, operating at 125.8 MHz. Chemical shifts are reported in ppm downfield from tetramethylsilane, and coupling constants are reported in Hz. ^77^Se-NMR spectra were on the same instrument operating at 95.4 MHz, using Me_2_Se (in C_6_D_6_) as external reference (−1 ppm) [24]. Resonance and structural assignments were based on the analysis of coupling patterns, including the ^13^C-^1^H coupling profiles obtained in bidimensional heteronuclear multiple bond correlation (HMBC) and heteronuclear single quantum coherence (HSQC) experiments, performed with standard pulse programs.

### 3.3. Caffeine Selenation Mediated by WR under Microwave Irradiation

#### 3.3.1. General Method for Optimization of Experimental Conditions

WR (103–206 µmol) was added to a solution of caffeine (50 mg, 257 µmol) in a suitable solvent (3 mL) (Table 1). The microwave vial was sealed, and the resulting solution was stirred at 175–300 W from 5 to 180 min (Table 1). Following solvent evaporation at reduced pressure, the mixture was purified by PTLC on silica (9/1 dichloromethane/methanol).

#### 3.3.2. Optimized Conditions

WR (83 mg, 156 µmol) was added to a solution of caffeine (50 mg, 257 µmol) in dry *p*-xylene (3 mL). The microwave vial was then sealed and the resulting solution was stirred, for 3 h, under microwave irradiation (300 W) reaching 170 °C (maximum temperature). Following cooling to room temperature, the *p*-xylene was evaporated under reduced pressure. The residue was dissolved in dichloromethane and purified by PTLC on silica (9/1 dichloromethane/methanol), affording *1,3,7-trimethyl-6-selenoxo-6,7-dihydro-1H-purin-2(3H)-one* (**2**, 6-selenocaffeine) as an yellow solid (28 mg, 42%). Purity 98% (HPLC-DAD); Rf = 0.66 (9/1 dichloromethane/methanol); mp 225–227 °C; ^1^H-NMR (500 MHz, DMSO-d_6_): 8.35 (1H, s, H8), 4.10 (3H, s, C12-H_3_), 3.75 (3H, s, C10-H_3_), *c.a.* 3.38 (partially obscured by water signal, C11-H_3_); ^13^C-NMR (125.8 MHz, DMSO-d_6_): 175.8 (C6), 149.1 (C2), 147.4 (C8), 143.8 (C4), 121.2 (C5), 37.4 (C10), 35.8 (C12), 30.3 (C11); ^77^Se NMR (95.4 MHz, DMSO-d_6_): 600.5 (C=Se); IR (KBr): υ_max_ = 1697 (C2=O), 1035 (C6=Se) cm^−1^; MS (ESI+) *m/z*: 261 [M(^82^Se)H]^+^ (20), 260 [M(^81^Se)H]^+^ (10), 259 [M(^80^Se)H]^+^ (100), 258 [M(^79^Se)H]^+^ (5), 257 [M(^78^Se)H]^+^ (35), 256 [M(^77^Se)H]^+^ (15), 255 [M(^76^Se)H]^+^ (17); HRMS (EI) *m/z*: [M(^82^Se)]^+^ calc. for C_8_H_10_ON_4_^82^Se (260.00161) found 260.00210, [M(^81^Se)]^+^ calc. for C_8_H_10_ON_4_^81^Se (259.00479) found 259.00823, [M(^80^Se)]^+^ calc. for C_8_H_10_ON_4_^80^Se (258.00144) found 258.00069, [M(^79^Se)]^+^ calc. for C_8_H_10_ON_4_^79^Se (257.00557) found 257.00193, [M(^78^Se)]^+^ calc. for C_8_H_10_ON_4_^78^Se (256.00221) found 256.00302, [M(^77^Se)]^+^ calc. for C_8_H_10_ON_4_^77^Se (255.00041) found 255.00186, [M(^76^Se)]^+^ calc. for C_8_H_10_ON_4_^76^Se (254.00412) obtained 254.00344.

### 3.4. Uracil Selenation Using Optimized Conditions

WR (83 mg, 156 µmol) was added to a solution of uracil (29 mg, 257 µmol) in dry *p*-xylene (3 mL). The microwave vial was then sealed and the resulting solution was stirred, for 3 h, under microwave irradiation (300 W) reaching 170 °C (maximum temperature). Following cooling to room temperature, the precipitate was filtered, dissolved in THF and purified by column chromatography on silica (*n*-hexane/ethyl acetate), affording *4-selenoxo-3,4-dihydropyrimidin-2(1H)-one* (6-selenouracil) (7.9 mg, 16%); ^1^H-NMR (500 MHz, DMSO-d_6_): 7.40 (1H, d, *J *= 5.8, H6), 6.47 (1H, d, *J* = 5.8, H5), ^1^H-NMR similar to that reported in literature [41];^13^C-NMR (DMSO-d_6_): 191.7 (C4=Se), 148.4 (C2=O), 137.9 (C6), 116.4 (C5); ^77^Se-NMR (95.4 MHz, DMSO-d_6_): 679.0 (C6=Se); IV (KBr) υ_max_: 1695 (C2=O), 1093 (C4=Se) cm^−1^. 

### 3.5. Caffeine Selenation under Conventional Heating

To a solution of caffeine (50 mg, 257 µmol) in dried *p*-xylene (3 mL) it was added WR (83 mg, 156 µmol). The mixture was stirred for 3 h at 170 °C. Upon cooling to room temperature the solvent was evaporated. The residue was dissolved in dichloromethane and purified by PTLC on silica (9/1 dichloromethane/methanol), affording *1,3,7-trimethyl-6-selenoxo-6,7-dihydro-1H-purin-2(3H)-one* (**2**, 6-selenocaffeine, 1.4 mg, 2%). Physical and spectroscopic data are identical to the one described above.

### 3.6. DPPH Free Radical Scavenging Assay

The free radical scavenging activity of **2** was evaluated using the DPPH assay [42]. 6-selenocaffeine (**2**) was dissolved in absolute ethanol and mixed with DPPH solution (in absolute ethanol; final concentration of 75 μM). The mixture (final volume = 700 μL) was incubated for 30 min in the dark at room temperature and the absorbance was monitored at 517 nm, against a blank containing the same concentration of 6-selenocaffeine. Ascorbic acid (100 μM, positive control) and caffeine (5, 10 and 15 mM) were also evaluated as described. Two independent experiments were carried out, each comprising duplicate tests per sample.

### 3.7. Cell Culture

The non-tumor human mammary MCF10A cell line and the human breast cancer MCF7 cell line were obtained from ATCC and DSMZ, respectively. MCF10A cells were cultured in DMEM/F12 medium, containing 5% horse serum, 100 U/mL penicillin, 0.1 mg/mL streptomycin, 0.01 mg/mL insulin, 0.5 μg/mL hydrocortisone, 100 ng/mL cholera toxin, and 20 ng/mL human EGF. MCF7 cells were cultured in DMEM medium, containing 10% FBS, 100 U/mL penicillin, 0.1 mg/mL streptomycin and 0.01 mg/mL insulin. Cells were kept at 37 °C, under a humidified atmosphere of 5% CO_2_-in air.

### 3.8. Cytotoxicity Evaluation

The MTT assay was performed using two different cell lines, MCF10A (non-tumor human mammary cells) and MCF7 (human breast cancer cells), according to Fernandes *et al. *[38]. For MCF10A and MCF7, 4.0 × 10^3^ cells and 6.0–6.5 × 10^3^ cells, respectively, were inoculated in 200 μL of culture medium per well in 96-well plates and incubated at 37 °C under a 5% CO_2_ atmosphere. The cells were grown for 48 h and then exposed to the test compound for a 24 h-period. Afterwards, the MTT assay was performed as previously described [33,36,38]. Eight replicate cultures were carried out.

### 3.9. Statistical Analysis

The differences in mean values of the results observed in cell cultures with different treatments were evaluated by the Student’s t-test, after verifying the normality using the Kolmogorov-Smirnov test. For non-normal variables the Mann-Whitney test was used. The results from the DPPH assay were analysed by the Student’s t-test. All analyses were performed with the SPSS statistical package (version 17, SPSS Inc. Chicago, IL, USA).

## 4. Conclusions

A novel organoselenium derivative, 6-selenocaffeine, was efficiently prepared from caffeine by an emerging microwave methodology. The additional example of the successful selenation of uracil, using the optimized experimental conditions, anticipates the general character of this synthetic methodology. 6-selenocaffeine was fully characterized by NMR and mass spectrometry and revealed to have improved antioxidant activity when compared with caffeine, along with low cytotoxicity potential towards normal mammary cells. Moreover, the results achieved with the seleno-derivative in breast cancer cells pointed out to differential roles in the modulation of the cytotoxicity of anticancer agents, whereas no cytotoxicity was observed (under the tested conditions) for 6-selenocaffeine alone. Indeed, this report constitutes a further step in the development of novel organoselenium compounds towards their potential applications in cancer therapy. 

## Supplementary Materials

Supplementary materials can be accessed at: http://www.mdpi.com/1420-3049/18/5/5251/s1.
