# Supplementary material for: Synthesis and Biological Activity of 6-Selenocaffeine: Potential Modulator of Chemotherapeutic Drugs in Breast Cancer Cells
Source: Molecules. 2013 May 8;18(5):5251–64. doi: 10.3390/molecules18055251 (PMC6270583; doi:10.3390/molecules18055251)
Supplement: Supplementary file 1 [file molecules-18-05251-s001.pdf]

Supplementary Materials

Figure S1. ^{1}H-NMR spectrum of 6-selenocaffeine, recorded in DMSO-d_6.

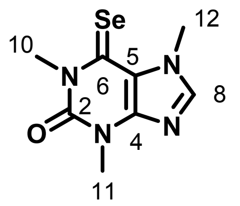

(2)

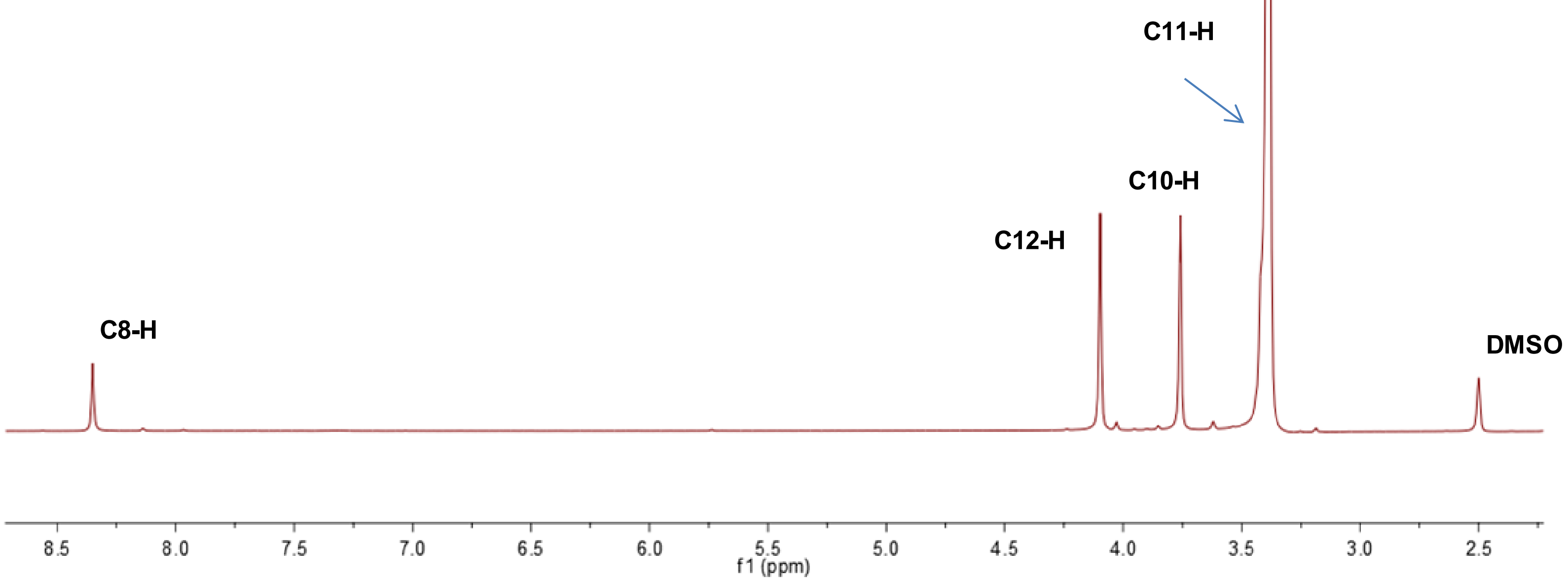

Figure S2. ^{13}C-NMR spectrum of 6-selenocaffeine, recorded in DMSO-d_6.

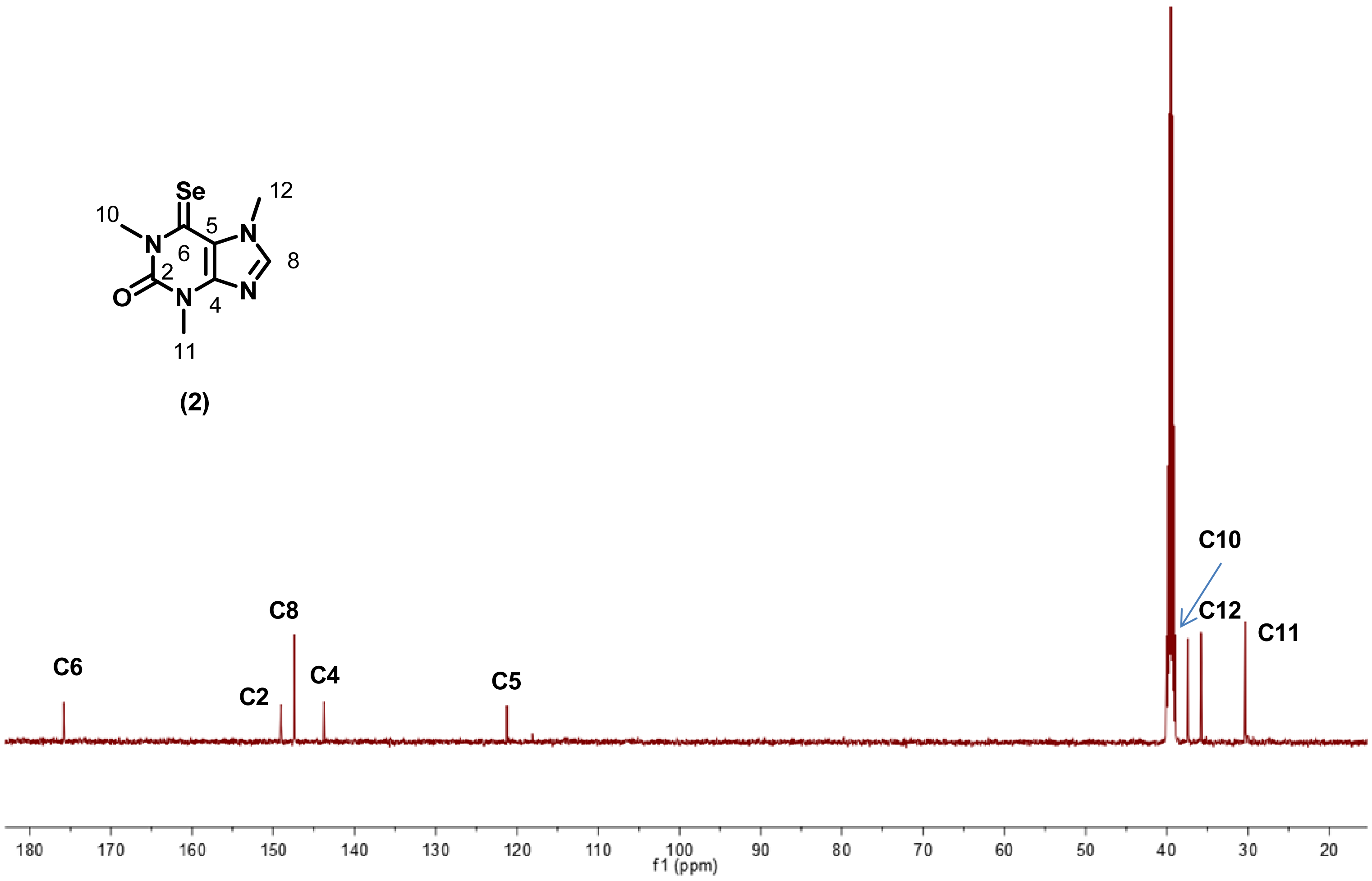

Figure S3. HSQC spectrum of 6-selenocaffeine, recorded in DMSO-d_6.

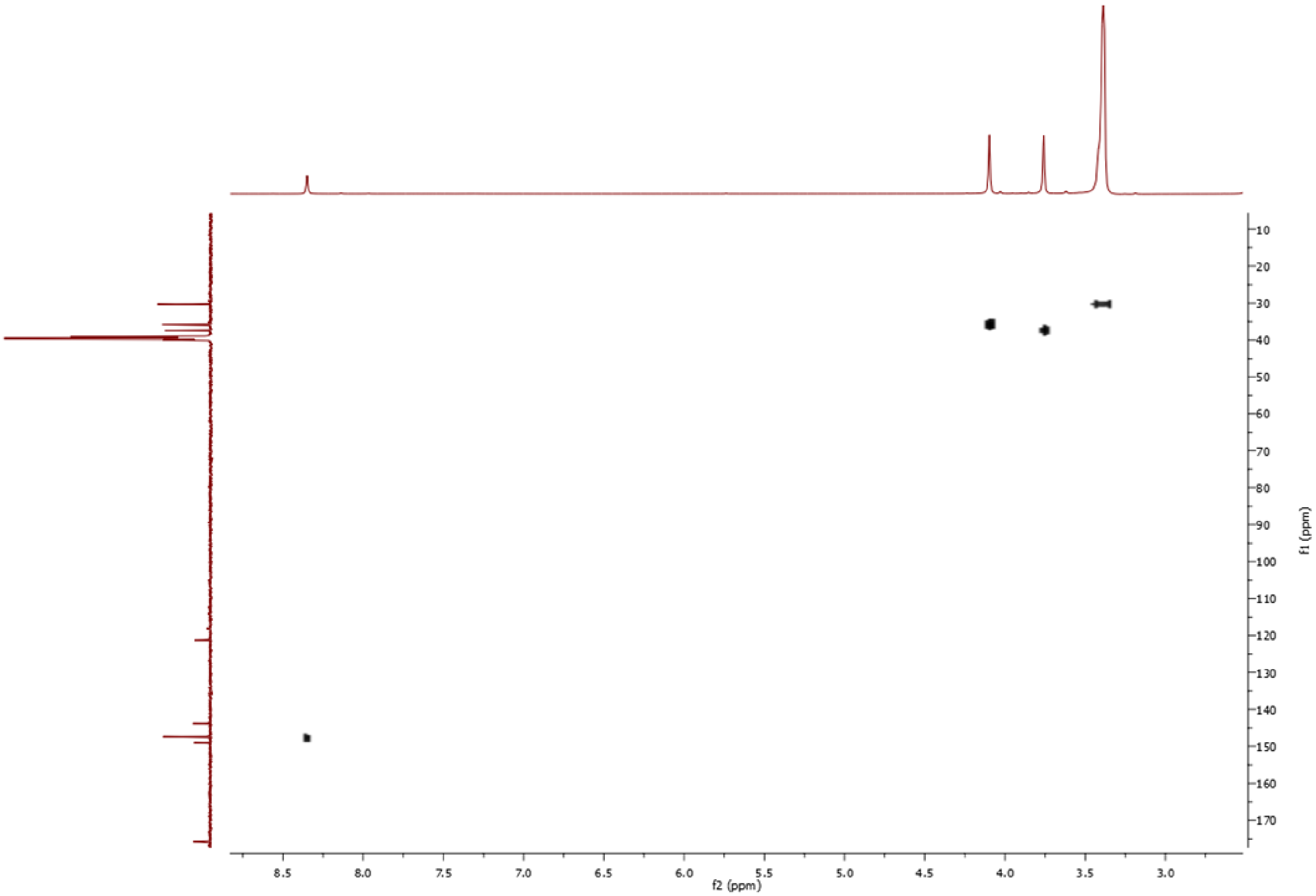

Figure S4. ^{77}Se-NMR spectrum of 6-selenocaffeine, recorded in DMSO-d$_6$.

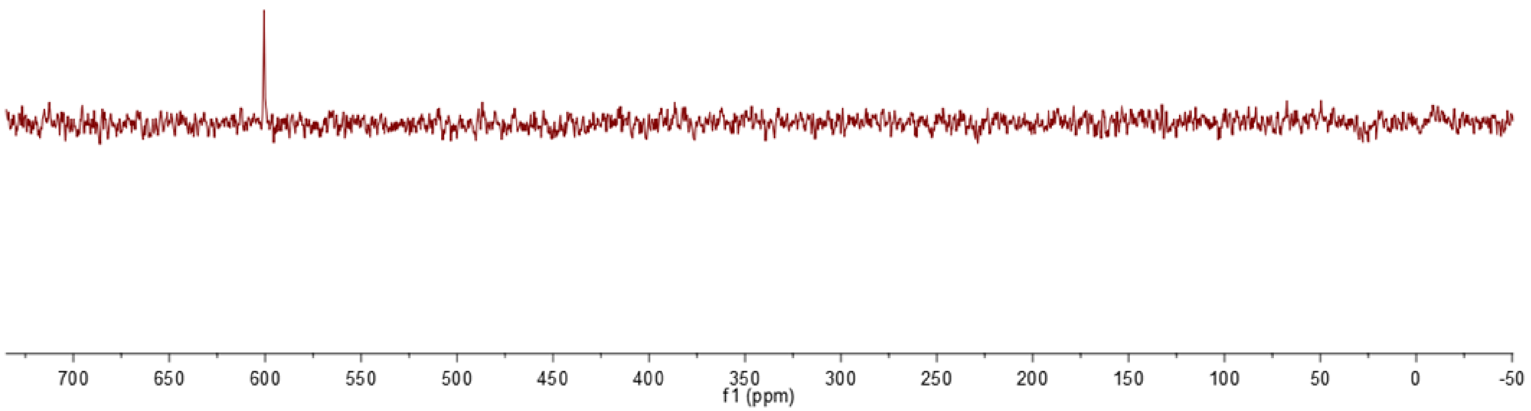

Figure S5. Mass spectrum HRMS (EI) of 6-selenocaffeine.

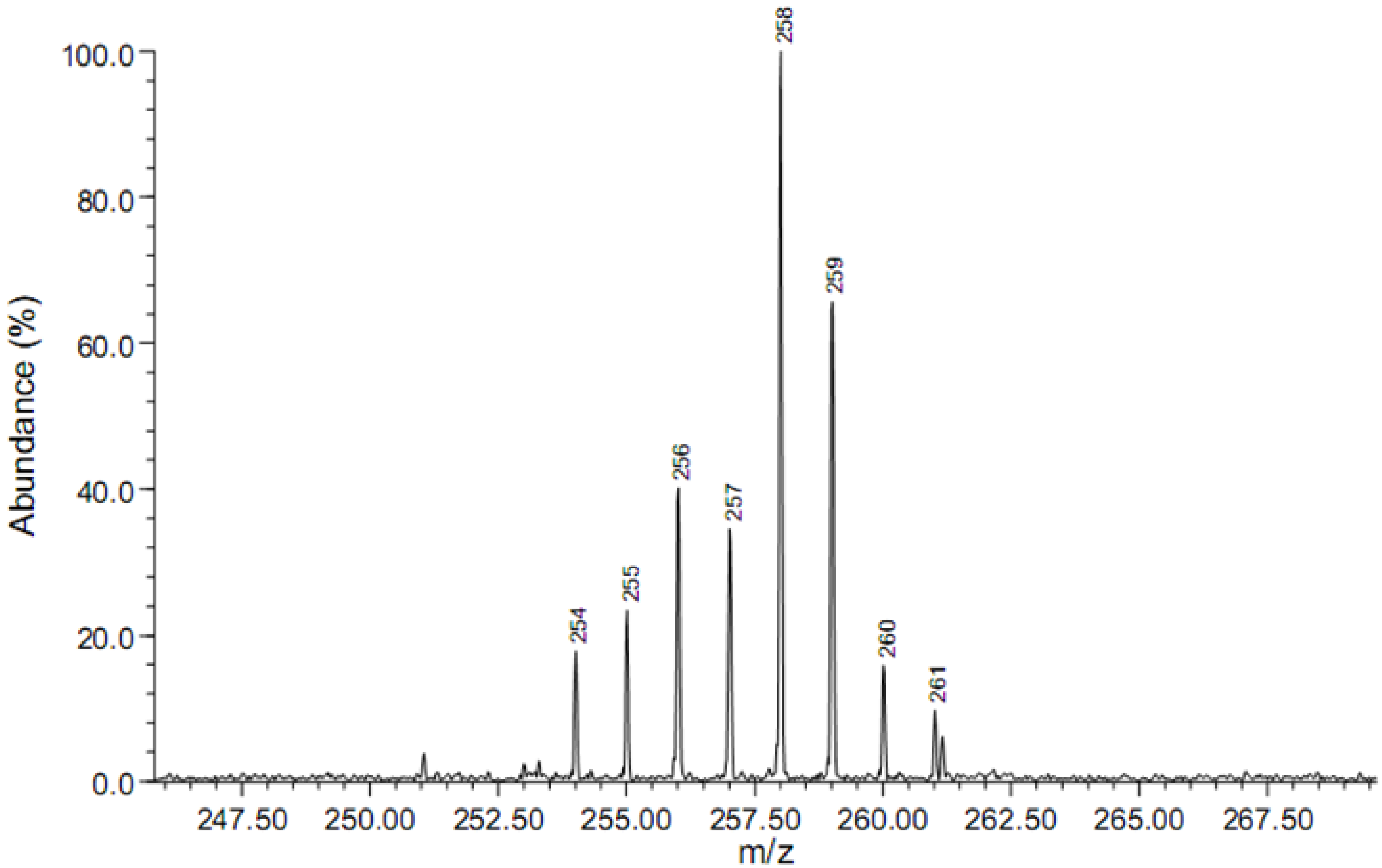

Figure S6. HPLC-DAD chromatogram of 6-selenocaffeine.

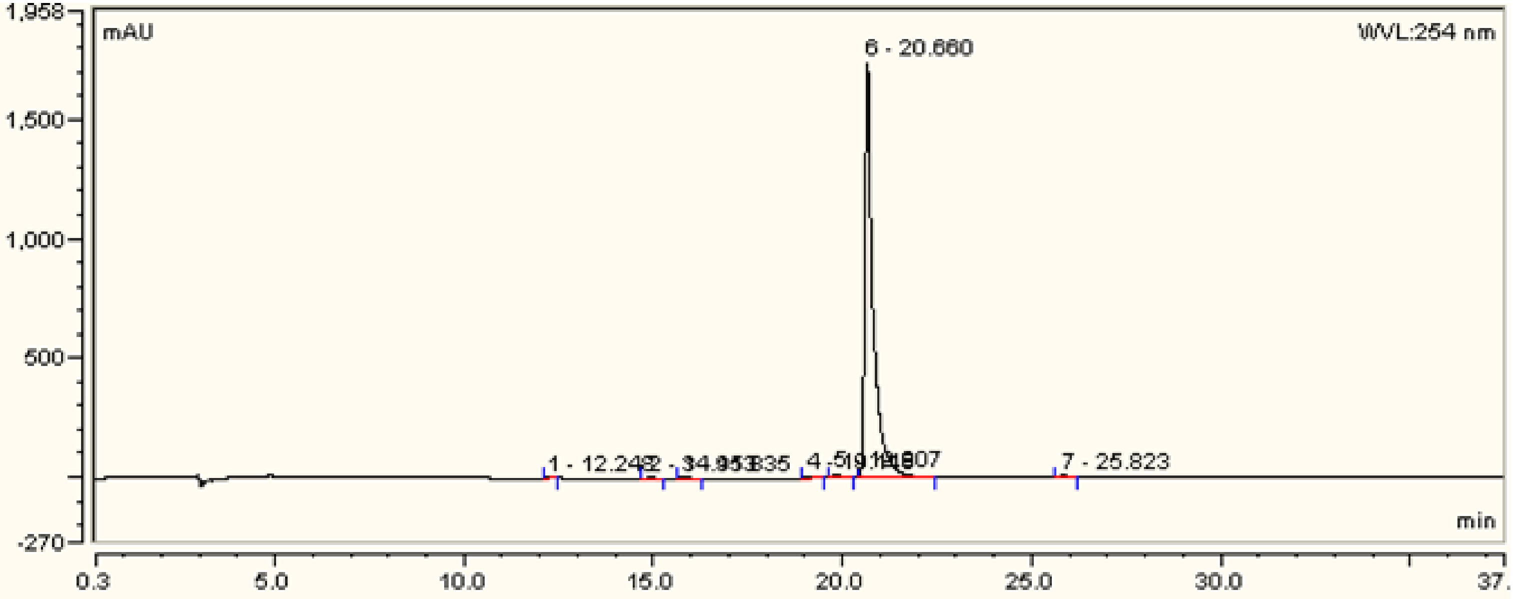

A	B	C	D	E	F	G	H	I
**No.**	**Peakname**	**Ret.Time min**	**Area mAU*min**	**Amount**	**Type**	**Height mAU**	**Rel.Area %**	**Resolution**
1	n.a.	12.248	0.7864	n.a.	BMB*	4.266	0.18	10.27
2	n.a.	14.913	0.2080	n.a.	BMB*	1.312	0.05	2.75
3	n.a.	15.835	0.3865	n.a.	BMB*	1.368	0.09	8.72
4	n.a.	19.118	1.4783	n.a.	BMB*	6.886	0.33	2.41
5	n.a.	19.807	3.5079	n.a.	BMB*	16.844	0.79	3.01
6	n.a.	20.660	436.0293	n.a.	BMB	1738.476	98.02	15.96
7	n.a.	25.823	2.4300	n.a.	BMB*	10.076	0.55	n.a.
**Total:**			**444.8264**	**0.0000**		**1779.228**	**100.00**	
